# Antigiardial Effect of Kramecyne in Experimental Giardiasis

**DOI:** 10.1155/2017/6832789

**Published:** 2017-12-13

**Authors:** Leticia Eligio-García, Elida Pontifez-Pablo, Salúd Pérez-Gutiérrez, Enedina Jiménez-Cardoso

**Affiliations:** ^1^Parasitology Research Laboratory, Hospital Infantil de México Federico Gómez, Dr. Márquez 162, Col. Doctores, Delegación Cuauhtémoc, 06720 Ciudad de México, Mexico; ^2^Biological Systems Department, UAM Xochimilco, Calzada del Hueso 1100, Col. Villa Quietud, Delegación Coyoacán, 04960 Ciudad de México, Mexico

## Abstract

A variety of drugs are used in giardiasis treatment with different levels of efficiency, presence of side effects, and even formation of resistant strains, so that it is important to search new only-one-dose treatments with high efficiency and less side effects. Kramecyne, an anti-inflammatory compound isolated from methanolic extract of* Krameria cytisoides*, does not present toxicity, even at doses of 5,000 mg/kg. The objective was to determine the antigiardial effect of kramecyne over* Giardia intestinalis in vitro* and* in vivo* and analyze the expression of genes ERK1, ERK2, and AK on kramecyne treated trophozoites by Real Time Polymerase Chain Reaction (RTPCR). The median lethal dose (LD_50_) was 40 *μ*g/mL and no morphological changes were observed by staining with blue trypan and light microscopy; experimental gerbil infection was eliminated with 320 *μ*g/Kg of weight. After treatment there were no differences between intestines from treated and untreated gerbils. Kramecyne did not have significant effect over ERK1 and AK, but there are differences in ERK2 expression (*p* = 0.04). Results show antigiardial activity of kramecyne; however the mode of action is still unclear and the evaluation of ultrastructural damage and expressed proteins is an alternative of study to understand the action mechanism.

## 1. Introduction

*Giardia intestinalis* is the most frequent parasite reported in children and the most frequently found protozoa in water samples [1, 2]; it mainly affects children and immunosuppressed people; this protozoan presents two interconvertible forms: cyst and trophozoite. Infection is acquired by ingestion of water and food contaminated with cysts, which become trophozoites and colonize the small intestine by attachment to the epithelial microvillus [3, 4]. Some infected people present gastrointestinal symptomatology and in many cases the disease is completely asymptomatic and the patients act as carriers releasing big quantities of cyst to the environment and promoting the transmission of infection [5].

There are several pharmaceutical agents that are used in the treatment of the giardiasis including metronidazole [6], albendazole, tinidazole, and nitazoxanide [7]; some of them are administered by one dose and others (as metronidazole) need several doses along the day for two or three days. In refractory cases by resistance or reinfection the combination of two or more drugs can be necessary [8, 9]. All chemical agents produce collateral effects like nausea, metallic taste, yellow pigmentation of the skin, hepatic damage, inefficacy in the treatment, and resistance [10]. Actually research studies focused on the search of new and better drugs against* Giardia intestinalis* are made in endemic areas, follow strategies to develop giardicides, and reevaluate the characteristics of existent drugs [11].

Two major reasons to search new drugs are the increment of resistant strains and the need to give alternatives in the* Giardia *treatment with only one dose, high efficiency, and less side effects; natural medicine is a viable alternative.

Some natural products developed to increasing antigiardial activity are alcoholic extracts of ancestral plants used as natural medicine in Africa and in India an immunomodulated drug obtained from Pippali Rasayana produced up to 98% recovery from the infection [13]. In Cuba, the most studied natural product with giardiasic activity is the propoleum obtained from bees because of its flavonoids, getting up to 60% healing efficiency versus 40% of tinidazole; this product is easy to obtain and without side reactions [14].

**Figure 1:**
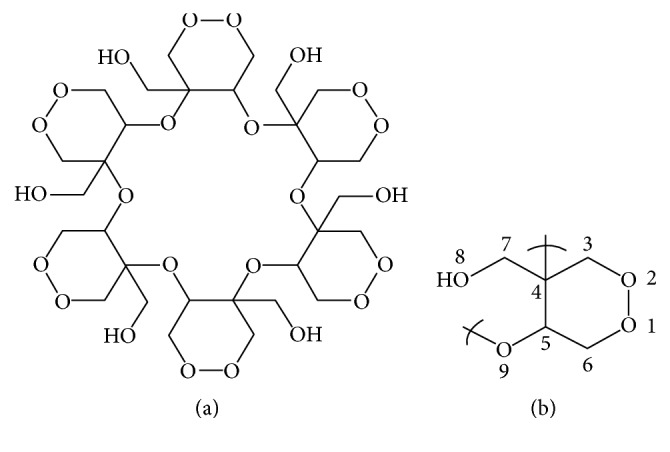
Chemical structure of (a) kramecyne (cyclic polymer) and (b) monomer [12].

In Metropolitan University of Mexico, a research team has isolated a methanolic extract from* Krameria cytisoides*, a plant collected in a central region of Mexico and frequently used to treat stomachache and other gastrointestinal affections [15]. The compound called kramecyne presents a chemical structure of a polymer of six monomers of cyclic peroxide (Figure 1), which was determined by Infrared (IR) spectrum, magnetic nuclear resonance (MNR), and mass spectrometry (MS) and elemental analysis (http://www.freepatentsonline.com/WO2013077719.html). This product exhibits anti-inflammatory activity and does not appear to cause cell damage even to 5000 mg/kg [12]. Kramecyne is suspected to have antiprotozoan activity and it has been evaluated for the ability to kill several protozoan parasites including* Giardia intestinalis*. Our preliminary studies that suggest antigiardial ability of kramecyne and to evaluate the mechanism of action of this compound we evaluate by molecular techniques some vital genes in its structure, differentiation, and metabolism that could probably be a target for action.

The objective of this work was to determine the therapeutical effect of kramecyne over* Giardia intestinalis* trophozoites* in vitro* and* in vivo* and analyze the expression of genes: ERK1, ERK2, and AK on treated trophozoites by Real Time Polymerase Chain Reaction (RTPCR).

## 2. Material and Methods

### 2.1. Effect* In Vitro*

#### 2.1.1. Kramecyne Solution

Kramecyne, a compound isolated from* Krameria cytisoides*, was obtained and kindly provided by Dr. Salúd Pérez Gutierrez from Biological Systems Department of UAM Xochimilco. From original extract of kramecyne, solutions diluted in TYI-S-33 media [16] were prepared to obtain 0, 20, 40, 80, 160, and 320 *μ*g/mL.

#### 2.1.2. Reference Strains of* Giardia*

The strains of* Giardia intestinalis* used in this study were P-I (ATCC® 30888, 1971) and WB (ATCC® 30957, 1983), both isolated from infected symptomatic patients, the first one in USA and the last one in Afghanistan. Both were cultured to 37°C and 5% of CO_2_ in TYI-S-33 media complemented with fetal bovine serum 10% and bovine bile 1.0 mg/mL.

#### 2.1.3. Median Lethal Dose (LD_50_) Determination

The concentration that inhibits 50% of parasite growth was determined as follows. Axenically cultured* Giardia *trophozoites (P-I and WB strains) were collected in logarithmic growth phase of a culture incubated at 37°C for 72 h, the tubes are placed on ice bath for 10 minutes, and then concentration of parasites was adjusted to 1 × 10^6^/0.5 mL by counting with Neubauer chamber. Trophozoites suspension was put to each tube containing 10 mL of medium and specific concentrations of the extract (0, 20, 40, 80, 160, and 320 *μ*g/mL) and incubated for 72 h at 37°C. After this time the suspensions are placed back on ice for 5 minutes and centrifuged at 2500 ×g during 10 min; the number of viable trophozoites stained with trypan blue was determined by counting in Neubauer chamber. In addition, the same assay was performed using the P-I strain of* Giardia* and a modified variant resistant to albendazole P-I(r); both incubated each concentration used for kramecyne but instead with albendazole (0, 20, 40, 80, 160, and 320 *μ*g/mL), the test was carried out under the same conditions of time and temperature and the harvest and count was carried out in the same manner as mentioned above. Each count was performed in triplicate and LD_50_ value was determined [17].

### 2.2. Effect* In Vivo*

#### 2.2.1. Cyst Purification

From feces obtained from children, Faust coproparasitoscopico analysis (CPS) was carried out for determining the presence of* Giardia *cysts. Feces were fully diluted with distilled water and filtered through gauze and the filtrate was stored at 4°C [18].

Cysts were purified from filtered human feces by sucrose gradients (Sigma S9378) as follows: 5 mL of stool filtrate was completed to 14 mL with distilled water, spun at 400 ×g for 5 minutes, and resuspended in distilled water. A volume of 1 M sucrose with density 1.11 g/ml was added and centrifuged at 400 ×g for 5 minutes. The cysts were observed in sucrose-water interface; the presence of parasitic structures in layer was verified by microscopic observation. Cysts were washed and resuspended in 1 mL of distilled water to be counted in a Neubauer chamber [19].

#### 2.2.2. Experimental Infection in Gerbils* (Meriones unguiculatus)*

Mongolian gerbils that were 21 days old were randomly transferred to separate cages to form five groups of 6 gerbils weighing between 15 and 25 g, parasite-free determined by CPS. Gerbils were inoculated in an intragastric way with 1 × 10^6^ purified cysts. After 5 days, stool samples were collected daily for CPS examination, until gerbils were positive to* Giardia *cysts in feces. They were kept under animal room conditions with food and water ad libitum all the time [20]. Additionally two more groups were included; as controls, in the first one, all the animals were kept free of infection and in the second one, the gerbils were infected but they were not treated with kramecyne.

#### 2.2.3. Kramecyne Administration

When* Giardia *cysts were observed in feces, kramecyne was administered orally at concentrations of 40, 320, 450, 500, and 750 *μ*g/Kg of weight during 5 days. CPS analysis was made daily until day 26 after inoculation. Animals were sacrificed by cervical dislocation and the entire small intestine was resected and opened longitudinally; the gut contents were scraped with a scalpel into a disposable Petri dish containing PBS buffer. The content of Petri dish was observed by microscopy to search* Giardia* trophozoites.

### 2.3. Molecular Study

#### 2.3.1. Kramecyne Solutions

 Kramecyne solutions were prepared from the original extract of kramecyne, to obtain final concentrations of 0, 20, 40, 80, and 160 *μ*g/mL.

#### 2.3.2. Growth Inhibition Assay

*Giardia *trophozoites were collected in logarithmic growth phase as mentioned earlier to obtain a concentration of 1 × 10^6^/0.5 mL by counting with Neubauer chamber. This parasite suspension was added to each tube with 2.5 mL of the culture medium and the specific concentration of the extract (0, 20, 40, 80, and 160 *μ*g/mL, resp.) in triplicate. A comparison control using 1 × 10^6^ trophozoites and 200 *μ*g albendazole was included. Treated cultures were incubated for 24 h at 37°C.

#### 2.3.3. RNA Extraction

After incubation, treated trophozoite suspension was used to obtain total RNA using* RNAqueous kit (Cat. # AM1912, Ambion) *according to manufacturer instructions. Two-hundred microliters of lysis-binding buffer was added to each sample in order to disrupt the cells. Samples were then mixed by vortex and allowed to stand at room temperature for 5 min with 200 *μ*L of 64% ethanol; then it was passed through a column and two washes were done to remove contaminating salts; total RNA was eluted using 30 *μ*L of heat elution buffer and the eluate was transferred to a new microcentrifuge tube. Eluted RNA was stored at 4°C after DNase treatment to degrade contaminating DNA.

#### 2.3.4. cDNA Synthesis

cDNA was synthesized by reverse transcription adding 10 *μ*L of RNA using the amplification program: 37°C for 60 min, 70°C for 10 min, 22°C for 1 min, and 4°C. DNA concentration was estimated by measuring the absorbance at 260 nm (using the relationship that an *A*_260_ of 1.0 = 50 *μ*g/ml pure dsDNA). Integrity was observed in 1% agarose gel electrophoresis.

#### 2.3.5. Design of Oligonucleotides

Oligonucleotides were designed using the software primer 3 [21] and based on the sequence of genes reported in the GenBank (https://www.ncbi.nlm.nih.gov/genbank/): extracellular signal-regulated kinase 1 (ERK1, gi|25527267): ERK1F (5′-CAGAATTGCGTCGGAT-3′) and ERK1R (5′-AGGCTCGTCAGACTCATCGT-3′); extracellular signal-regulated kinase 2 (ERK2, gi25527281): ERK2F (F5′-AATCCCAACAAGCGACTGAC-3′) and ERK2R (5′-GCGGATCTCCTTCTTTTTCC-3′); adenylate cyclase (AK, gi|639927): AKF (5′-TTGGCTTTTGGATGGATTTC-3′) and AKR (5′-CTTCTCTGTCGTCGGTCCTC-3′). To test the capacity of amplification, a polymerase chain reaction (PCR) was made from 100 ng of cDNA with PCR Master Mix (Promega) and the amplification program of initial denaturation at 94°C followed by 30 cycles at 94°C for 30 s, an alignment step at 50–60°C for 30 sec, an extension step at 72°C for 30 s, and a final extension of 72°C for 5 min. The amplified products were resolved by 1.5% agarose gel electrophoresis, which was stained with ethidium bromide and visualized under UV illumination.

#### 2.3.6. qRT-PCR

The mixture was prepared by using Maximum SYBR Green qPCR Master Mix (Thermo Fisher Scientific, USA), in a 15 *μ*L reaction mix, according to manufacturer instructions: Master Mix 1x, 0.1 nM of ROX solution, 0.3 *μ*M of each primer, and 300 ng of cDNA. Subsequently the reaction mixture was distributed into optical tubes and put in the thermal cycler (Agilent Technologies Stratagene, USA) with following two-step cycles protocol conditions: initial denaturation 10 minutes at 95°C, followed by 40 cycles at 95°C for 15 s and an alignment at 60°C for 1 min over final extension 95°C per 1 min and 60°C for 30 seconds (ERK1 and ERK2). For AK the program was the same except for alignment temperature that was 56°C.

### 2.4. Statistical Analysis

LD_50_ was determined graphically plotting a simple graphic representation of the concentration dose of kramecyne and % of viability after incubation. Value of 50% was obtained by data correlation taking into account the average of the values performed in triplicate. For results of RTPCR the value of *p* was determined for a test of significance *Z* with a significance level of 0.05.

### 2.5. Ethical Considerations

This study respects the copyright international. All involved people have been notified and they agree. Recollection of plants is according to norms of biodiversity conservation and we follow the NOM-062-ZOO-1999 of safety management of animals. It had been approved by local ethical and biosecurity committee of the institution.

## 3. Results and Discussion

In this study the antigiardial effect of kramecyne on* Giardia* trophozoites growth and the expression of some vital genes for the structure, differentiation (ERK1 and ERK2), and metabolism (AK) of* Giardia intestinalis* was determined.

Figure 1 exhibits the structure of kramecyne, molecule obtained from the* Krameria cytisoides* methanolic extract and characterized in that it is a cyclic polymeric peroxide which is formed by a monomer which is bonded by atoms 4 and 9 (http://www.freepatentsonline.com/WO2013077719.html). This molecule was studied to evaluate the capacity to inhibit* Giardia *growth.

**Figure 2:**
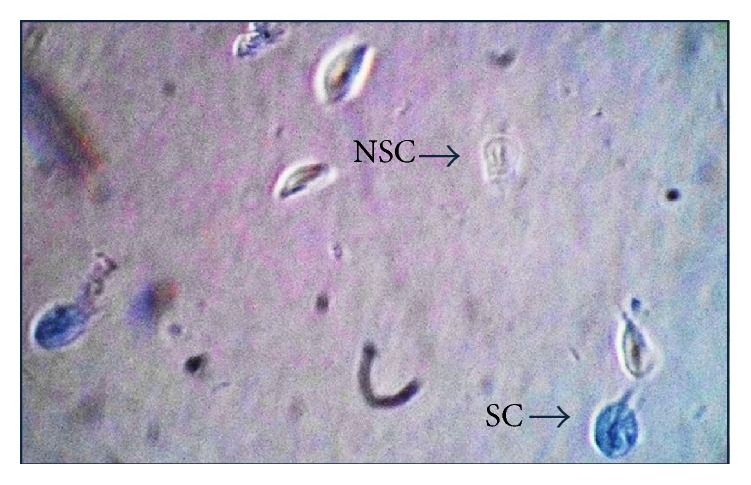
Trophozoites after treatment with 80 *μ*g/mL of kramecyne stained with trypan blue dye. Preparation allows differentiating dead cells (in blue; SC) and transparent living cells (NSC). No structural damage is observed caused by contact with kramecyne. Olympus BH (40x).

After treatment of axenic culture of* Giardia *with different concentrations of kramecyne, trypan blue staining allowed counting cells and also determining the proportion of viable and nonviable cells; Figure 2 shows blue-stained cells, indicating that the membrane of a nonviable cell has been permeated and allowed to enter the dye; in addition the staining allows observing that trophozoites did not appear to undergo structural modification by action of kramecyne; trophozoites did not lose their size and shape and did not show damage in nucleus structure, although ultrastructural studies are necessary to determine damage in a more specific way.

**Figure 3:**
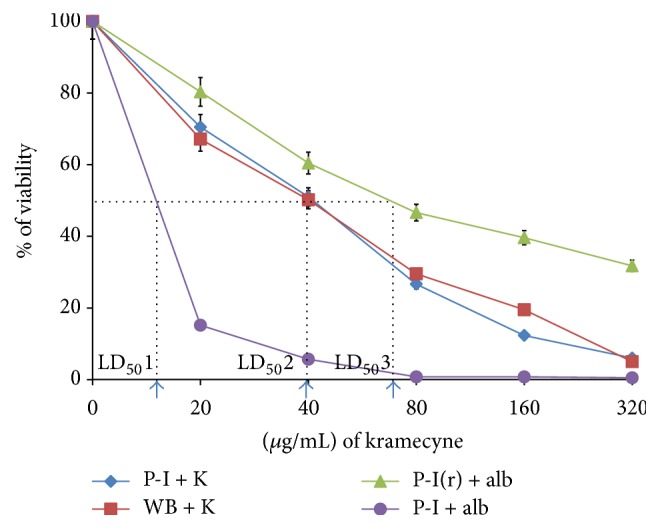
Results of effect* in vitro*, percentage of inhibition of two strains (P-I and WB ) of* Giardia intestinalis* axenically cultured treated with different concentrations of kramecyne (K), LD_50_2 value = 40 *μ*g/mL for both strains. Percentages of inhibition of P-I and P-I(r) are also observed (LD_50_1 = 12 and LD_50_3 = 70 *μ*g/mL, resp.).

LD_50_ was observed at concentration of 40 *μ*g/mL, in both P1 and WB strains, which means that the fifty percent of* Giardia *trophozoites are killed at this concentration; both reference strains exhibited similar behavior when cultured with a kramecyne concentrations gradient (Figure 3). These results were compared with the effect of albendazole in the PI strain and a strain resistant to albendazole; although the LD_50_ is much lower than the kramecyne value, its homologue resistant to this drug offers a LD_50_ value of 70 *μ*g/mL, and the presence of resistant strains in human infections requires administering higher doses of drugs and for longer period of time, which justifies the use of more efficient alternatives. Several studies have been conducted to evaluate antiparasitic properties of plant extracts against* Giardia intestinalis*; Ponce-Macotela et al. in 2006 [22] studied the effect of oregano* (Lippia berlandieri)* ethanolic extracts against* Giardia* trophozoites at concentrations ranging from 58 to 588 *μ*g and demonstrated ultra-structural lesion in trophozoites, losing shape, size, and damage to the nucleus. Similar studies have been reported to different natural extracts of plants;* Yucca baccata* exhibited effective antigiardial activity in the proximal segment similar to metronidazole [23]. The dichloromethane fraction of peppermint presented antigiardial activity* in vitro* with an IC(50) of 0.75 *μ*g/mL causing several alterations on plasma membrane surface of the parasite and inhibited the adhesion of* G. intestinalis* trophozoites [24], while another study concluded that water soluble extracts of blueberries can kill* G. intestinalis* trophozoites and modify the cell morphology [25].

*In vitro* model was performed in triplicate to confirm the repeatability; this procedure is made in a controlled environment, so it was possible to determine the LD_50_ value.* In vivo* study showed an efficacy value on giardiasis elimination of infected gerbils; the results at different concentrations are represented in Table 1. Concentrations used in this experiment were determined from the value of LD_50_ which was 40 and up to a concentration of 750 *μ*g/Kg of weight. Experimental groups of six gerbils were observed to obtain that, with 320 *μ*g/Kg of weight, we obtained 80% of efficiency to eliminate the experimental infection, but at 450, 500, and 750 *μ*g/Kg of weight this efficiency is lower (60%); it means that just two of six gerbils got infection-free (Table 1). In the control group the uninfected gerbils kept without* Giardia* until the end of experiment, and the infected/untreated gerbils remained parasitized until the 26th day after inoculation. In this work and in many cases published before the results seem variable and determined by several factors as biological variability of animal model; this could be minimized by an increment in the number of animal specimens tested by group. After treatment with kramecyne the content of the intestine was analyzed microscopically to evaluate the presence of trophozoites and to determine visible alterations in the intestine; none of the observations presented changes or differences between intestines of treated gerbils respecting nontreated ones. The results obtained in vivo and in vitro present differences and they cannot be compared due to the conditions in which each of them is carried out. Results show antigiardial activity of kramecyne* in vitro and in vivo*; it suggests that this compound could have a potential value as therapeutic agent against* Giardia intestinalis* infections. But it is important to know the mechanism of action by which this compound acts on* Giardia*.

We analyzed three genes: adenylate cyclase (AK), corresponding to phosphorus-oxygen lyase activity protein on cyclic nucleotide biosynthetic process and intracellular signal transduction [26], and two members of the MAPK family: extracellular signal-regulated kinases 1 and 2 (ERK1 and ERK2), which play a critical role in trophozoite differentiation into cysts [27, 28].

**Figure 4:**
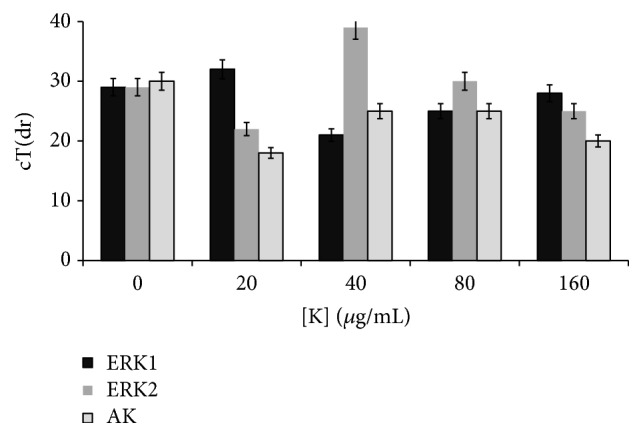
Graph of kramecyne concentration versus Ct values showing results of the expression determined by Real Time PCR reaction for the genes ERK1 (*z* = 0.5), ERK2 (*z* = 0.04), and AK (*z* = 0.99).

The Real Time PCR was performed to determine whether the expression of genes was affected or modified after treatment. When comparing ERK1 and AK expression levels against the untreated control, no statistical significance is observed. While with ERK2 significant difference (*p* = 0.04) is observed comparing with nontreated cells, and it is necessary to study more deeply into this gene and others related in order to establish if there are changes at this level in the trophozoite in contact with kramecyne (Figure 4). 

The antigiardiasic activity of kramecyne has been verified but its mechanism of action is still unclear; for this reason it is necessary to realize studies with other genes or at the level of proteins to know how and which molecules are involved in the action of kramecyne over trophozoites.

Kramecyne is a potent inhibitor of inOS, COX-2, NO, TNF-*α*, and IL-6 production at the transcriptional level in LPS-stimulated macrophages, which gives anti-inflammatory properties by involving ionic interchange of cell [29], and perhaps this feature is involved in the mechanism that causes the death of* Giardia *trophozoites in a similar way to the mechanism of omeprazole, which has been demonstrated to have potential for inhibiting giardial glycolytic enzyme triosephosphate isomerase (GiTIM) in a species-specific manner, with a deleterious effect only when targeting Cys 222 and killing* Giardia *trophozoites [30].

## Figures and Tables

**Table 1:** Results of effect *in vivo*. Efficiency of treatment of kramecyne over infected gerbils with experimental giardiasis. The chronological procedure in days is shown. Nontreated gerbils remain infected until day 26 and they were also euthanized.

Group	1 (*n* = 6)	2 (*n* = 6)	3 (*n* = 6)	4 (*n* = 6)	5 (*n* = 6)
Day 0	Intragastric inoculation Oral inoculation of 1 × 10^6^ purified cysts of *Giardia *into the gerbils				
Days 5–15	Infection CPS analysis searching *Giardia intestinalis *cysts				
Days 16–20	Treatment Oral inoculation of kramecyne (*μ*g/Kg of weight)				
	40	320	450	500	750
Days 21–26	Evaluation CPS to follow the infection status				
Day 26	Euthanasia of the gerbils at day 26				
Efficiency of treatment	Elimination of parasite				
	2/6	5/6	4/6	4/6	4/6
